# Laparoscopic ovarian tissue collection for fertility preservation in children with malignancies: a multicentric experience

**DOI:** 10.3389/fsurg.2024.1352698

**Published:** 2024-01-23

**Authors:** Federica Perelli, Giulia Fusi, Luca Lonati, Tommaso Gargano, Michela Maffi, Stefano Avanzini, Federico Palo, Maria Dolores Blanco Verdú, Agustín Del Cañizo López, Carmen Garrido Colino, Giulia Perucca, Girolamo Mattioli, Fabrizio Gennari, Mario Lima, Riccardo Guanà

**Affiliations:** ^1^ Department of Gynecology and Pediatrics, Azienda USL Toscana Centro, Florence, Italy; ^2^ Department of Pediatric Surgery, IRCCS Meyer Children’s Hospital, Florence, Italy; ^3^ Pediatric Surgery Unit, Regina Margherita Children’s Hospital, Turin, Italy; ^4^ Pediatric Surgery Unit, Sant’Orsola Children’s Hospital, Bologna, Italy; ^5^ Pediatric Surgery Department, IRCCS G. Gaslini, Genoa, Italy; ^6^ Pediatric Surgery Unit, Gregorio Maranon Children’s Hospital, Madrid, Spain; ^7^ Pediatric Radiology Unit, Great Hormond Street Hospital, London, United Kingdom

**Keywords:** fertility-sparing, cryopreservation, cancer survivors, pediatric cancer, ovarian tissue collection, minimally invasive surgery

## Abstract

**Introduction:**

Long survivors after childhood cancer are increasing thanks to oncological improvements. Their quality of life and fertility-sparing should be considered in the early phases of each oncological pathway. Cryopreservation of ovarian tissue removed before starting gonadotoxic therapies is the only fertility sparing procedure available for prepubertal children affected by cancer and it does not affect the timing of the start of the treatment.

**Materials and methods:**

The present study shows the surgical and clinical outcomes following laparoscopic ovarian tissue collection (LOTC) for a total of 311 patients aged between 0 and 17 years old from four different European Centers.

**Results:**

Only two major complications were reported according to the Clavien Dindo classification (0.6%).

**Discussion:**

LOTC can be considered a safe procedure.

## 1 Introduction

Survival after childhood cancer has improved over the past two decades and is now up to 80% considering all diseases. Nearly 75% of young cancer patients will be living more than 10 years after diagnosis (1). Moreover, the long-term survival rate of children undergoing hematopoietic stem cell transplant (HSCT) is constantly increasing. Improving in survival implies, on the other hand, an increase in morbidity for long term survivors.

Among all the late effects, infertility is reported as a major concern, especially in female cancer survivors due to the use of gonadotoxic treatments, like radiotherapy or chemotherapy, which may permanently impair reproductive functions.

Total body irradiation (TBI) and an older age at the time of HSCT can negatively affect the persistence of ovarian function and the onset of premature ovarian failure (POF).

When administered before puberty, TBI is less gonadotoxic, with 40%–60% of patients experiencing spontaneous recovery, vs. 10%–14% in post-pubertal girls (2).

Loss of ovarian function after chemotherapy with an alkylating agent (cyclophosphamide, busulfan) could result in both sterilization and endocrine function deficiency.

Due to all these factors the risk of infertility in patients undergoing a conditioning regimen for HSCT has been defined as higher than 80%.

Fertility preservation is a key component of POF management in children and should be considered for all young patients undergoing potentially gonadotoxic cancer treatments or at high risk for ovarian failure.

Cryopreservation of ovarian tissue is currently the main option available to preserve fertility in prepubertal patients who require cancer treatment but cannot postpone the beginning of chemotherapy.

The advantage of this technique is that it requires just a few days to be planned and performed, with a minimally invasive approach and, as the retrieval of ovarian tissue is not dependent on the menstrual cycle, no delay in treatments is required.

Furthermore, it has been demonstrated that laparoscopic ovarian tissue collection (LOTC) is a feasible technique even after abdominopelvic surgery (3). This technique allows the storage of a great number of primordial follicles that are relatively resistant to cryodamage (about 70%–80% survival) (4).

The aim of the study was to describe and compare the experience of four high-volume European pediatric Centers in terms of surgical outcomes, where oncological female patients at high risk for infertility are enrolled in the program of LOTC.

## 2 Materials and methods

Data from four European high-volume pediatric Centers were considered for this study; the hospitals involved were the Regina Margherita Children's Hospital in Turin, Italy (Center 1), the Sant' Orsola-Malpighi University Hospital in Bologna, Italy (Center 2), the Giannina Gaslini Children's Hospital in Genoa, Italy (Center 3) and the Gregorio Maranon Children's Hospital in Madrid, Spain (Center 4).

This was a retrospective study including girls and adolescents up to 17 years of age, who had undergone LOTC in the past two decades, before a highly gonadotoxic treatment for malignant diseases was initiated.

The indication for LOTC was established when the treatment planned included conditioning for autologous or allogeneic hematologic stem cell transplantation, high-dose chemotherapy or *in toto* abdominal irradiation or pelvic irradiation.

The clinical and demographic features of each patient were collected (Table 1).

**Table 1:** Demographic and clinical patients’ characteristics.

Demographic and clinical characteristics	Center 1 93 pts	Center 2 164 pts	Center 3 36 pts	Center 4 18 pts
Age at diagnosis mean (range)	11.12 (0–17)	11.12 (3–17)	12.1 (7–17.2)	4 Leukemia pts 4.86 (2–10) 14 other pts 13.7 (1–17)
Age at surgery mean (range)	13 (2.7–17)	13 (1.8–17)	15.5 (7–31)	5 (2–15)
Prepubertal at surgery *n* (%)	24 (25.8%)	42 (25.6%)	10 (27.8%)	15 (83.3%)
LOCS pathological indication *n* (%)	Sarcomas 13 (13.9%) Acute leukaemia 60 (64.5%) HL 10 (10.8%) NHL 10 (10.8%)	Sarcomas 47 (28.7%) Acute leukaemia, HL, NHL 93 (56.7%) CNS tumors 10 (6.1%) Wilms tumor 6 (3.6%) Others 8 (4.9%)	Sarcomas 12 (33.3%) HL, NHL 15 (41.7%) CNS tumors 4 (11.1%) Ovarian teratoma 1 (2.8%) BM aplasia, chronic granulomatous disease 4 (11.1%)	Sarcomas 2 (11.1%) HL 8 (44.4%) NHL 3 (16.7%) Leukemia 4 (22.2%) BM aplasia 1 (5.6%)

Qualitative data are *n* (%); quantitative data are median (range).

Pts, patients; HL, Hodgkin Lymphoma; NHL, Non-Hodgkin Lymphoma; CNS, central nervous system; BM, bone marrow.

The surgical outcomes were evaluated considering different variables: the duration of the hospital stay, the operative timing, the need for laparotomic conversion intraoperatively, and the postoperative complications were registered during a postoperative follow up of six months for each patient (Table 2).

**Table 2:** Surgical patients’ characteristics.

Surgical characteristics	Center 1 93 pts	Center 2 164 pts	Center 3 36 pts	Center 4 18 pts
Operative time (min) mean (range)	45 (35–55)	40 (35–45)	55 (45–65)	65 (55–75)
Type of ovarian sampling	Bilateral sampling 93 (100%)	Monolateral sampling 164 (100%)	Monolateral sampling 31 (86.1%) Bilateral sampling 5 (13.9%)	Monolateral sampling 18 (100%)
Intra/postoperative complications^a^ *n* (%)	1 Dindo 3 (1%)	0 (%)	0 (%)	1 Dindo 3 (5.6%)
Laparotomic conversion *n* (%)	0 (%)	0 (%)	1 (2.8%)	0 (%)
Number and type of trocars	Three 5 mm trocars	One 10 mm trocar, Two 5 mm trocars	One 12 mm trocar, Two 5 mm trocars	Three 5 mm trocars
Use of drainage	No drains	Routinely inserted	No drains	No drains
Hospital stays (postoperative days) median (range)	2 (0–5)	1 (1–2)	2 (2–3)	1 (0–1)

Qualitative data are *n* (%); quantitative data are median (range).

Pts, patients.

^a^ According to Clavien-Dindo classification (5).

The postoperative complications were evaluated according to the Clavien-Dindo classification (5).

In Center 1, surgeons collected tissue from both ovaries by performing a bilateral sampling. The surgical procedure consisted in a 3-trocars 5 mm laparoscopy, collecting 50% of tissue of each gonad (ovarian cortex biopsies) without tissue cauterization to optimize the viability (only for hemostatic purpose after tissue removal), or draining the pelvis.

Trocar position was the following: the zero degrees camera port was positioned in the navel and the two operative ports were placed in the right and left iliac fossa respectively.

The sampling was then submitted for histological examination to detect any neoplastic contamination and, if negative, frozen, following the slow freezing procedure and cryopreserved in liquid nitrogen.

A hemoglobin check was planned in the first post-operative day and discharge was planned on the same day or on the second postoperative day.

In Center 2, a monolateral sampling was performed and the harvesting technique also consisted of a three-portal laparoscopy with a 10 mm umbilical port for a zero degrees camera and two 5 mm trocars for forceps and hemostatic devices. Only one ovary was subjected to biopsy, based on the pre-operative pelvic ultrasound. Surgical steps were the stabilization of the ovary by means of grasping forceps, followed by cutting the tissue to be removed with a cold blade. The tissue was excised with atraumatic forceps in a single point. Then the piece was externalized from the navel and accurate hemostasis was carried out with a monopolar hook or bipolar forceps. At the end of the procedure, a drain was routinely left in place and removed in the first postoperative day, after performing a control blood count. Discharge took place on the first or second post-operative day.

In Center 3, both a monolateral and a bilateral sampling was performed based on a pelvic ultrasound performed pre-operatively to check the presence and the numerosity of follicles in each ovary. The operative technique involved the use of 3 trocars, one 12 mm umbilical port for a 30-degree camera and two operating trocars, 5 mm on left hand and 12 mm on right hand. Surgical steps included identification of both ovaries and evaluation of the bigger one (confirming the pre-operative ultrasound findings), then stabilization of the ovary and removal of approximately half of the parenchyma of only one ovary, except in cases where the preoperative ultrasound and the surgical evaluation did not demonstrate dimensional superiority of one ovary, for which ovarian tissue was collected from both ovarian parenchymas. The sample was retrieved in an endobag and externalized through the umbilical access. Accurate hemostasis was carried out with a monopolar hook or scissor, in some cases the use of a vessel sealer device was needed. Drainage was not routinely inserted.

In Center 4, a monolateral sampling was collected and for the procedure, three 5 mm trocars were used. Antibiotic prophylaxis with Cefazolin was administered and only one ovary was sampled. For the cortex removal, a careful dissection with scissors was performed, avoiding the medulla of the ovary, fimbriae and vessels. Throughout the procedure, every attempt was made to avoid coagulation because of the risk of decreasing the viability of the primordial follicles.

To control hemostasis a hemostatic agent was preferred. No drains were left in place. The sample was removed through the umbilical trocar without an endobag. A member of the biology department was present during the entire procedure to ensure the correct and rapid transport of the sample, once it had been removed. The tissue was transferred to the laboratory where it was analysed and prepared for subsequent cryopreservation.

### 2.1 Cryopreservation/pathologic evaluation

**Figure 1:**
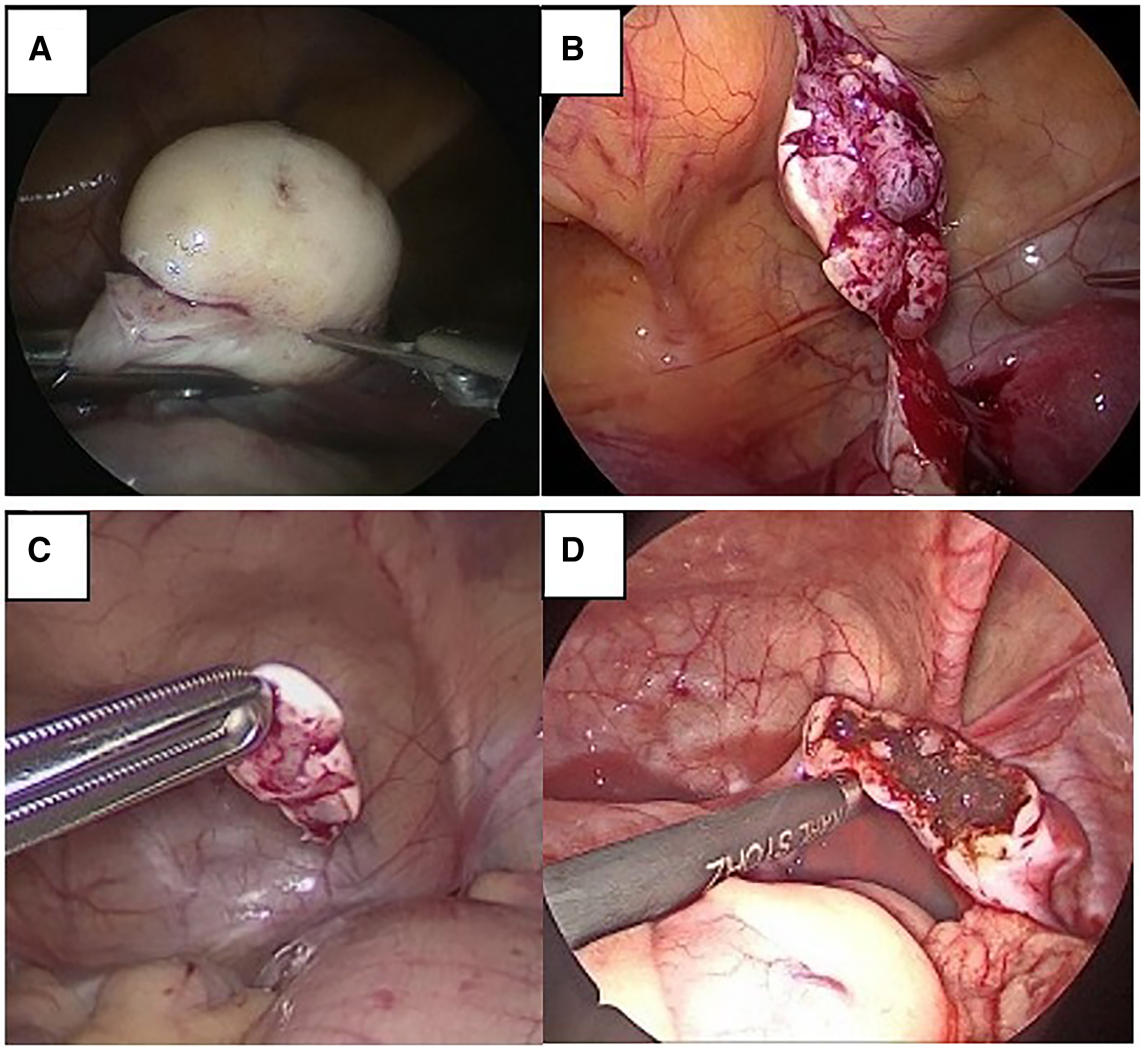
Intraoperative view of LOTC. (**A**) Cold scissors incision on ovarian parenchyma. (**B**) Laparoscopic removal of ovarian tissue. (**C**) Ovarian tissue fragment removed for cryopreservation. (**D**) Ovarian bed spared and coagulated with bipolar forceps for hemostatic effect.

The same cryopreservation process and pathologic evaluation criteria was applied to all the Centers involved in the study. The ovarian cortex is located a little deeper below the capsule and contains most of the primordial follicles. These primordial follicles represent the child's ovarian reserve. In the tissue cryopreservation technique, the cortex is dissected from the medulla and cut into cortical strips, washed to remove blood cells and then passed through a series of cryopreservation media (Figure 1). The processed strips are placed in cryovials and undergo a slow-freezing process to −40 °C. The tissues are then transported to a long-term reproductive tissue storage facility in liquid nitrogen. The intraoperative biopsy undergoes pathology evaluation for the presence and stage of ovarian follicles and the eventual presence of neoplastic cells. The quality of the ovarian tissue collected laparoscopically was directly proportional to the cold blade cut and inversely proportional to the surgical cauterization; the dimensions of the ovarian fragments collected were found to be satisfactory in all the Centers that provided the laboratory with a sufficiently represented quantity of three-dimensional tissue.

## 3 Results

### 3.1 Center 1

The experience in Center 1 in LOTC in children affected by neoplastic diseases, started in January 2002. A total of 93 procedures were performed up to September 2022, in patients undergoing HSCT or sterilizing chemotherapy. The median age at diagnosis was 11.12 years (range: 0–17.49 years) and the median age at the time of the procedure was 13 years (range: 2.7–17.3 years). Twenty-four patients were not pubertal at the time of surgery. Mean operative time was 45 min, sampling was always collected from both ovaries and no drain was left in place at the end of the procedure.

Primary pathologies were acute leukemia (60), Hodgkin's lymphoma (10), non-Hodgkin's lymphoma (10), sarcomas (13). All the patients in this series were candidates for HSCT.

One patient required re-laparoscopy in the first postoperative day due to incomplete hemostasis in one ovary, and subsequent anemization.

No malignant cells were identified at the histopathology evaluation.

### 3.2 Center 2

In Center 2, a total of 164 patients were treated from January 2002 to September 2022. Mean age at primary diagnosis was 11.12 years (range: 3–17.49 years). Mean age at the procedure was 13 years old (±4.19 aa, range 1.8–17.9), and 42 patients were not pubertal at the time of surgery. A pelvic ultrasound was performed pre-operatively to evaluate the follicular reserve and consequently which ovary would be sampled (monolateral sampling). The mean operative time was 40 min. The neoplastic pathologies were: leukemia/lymphoma (93), sarcomas (47), central nervous system tumors (10), Wilms' tumor (6), others malignancies (8).

Main indications for cryopreservation were the starting of chemotherapy and/or radiotherapy.

No post-operative complications were reported and patients were discharged on the first or second post-operative day.

### 3.3 Center 3

In Center 3, the program started in 2011, and a total of 36 patients were considered from May 2011 to September 2022. Mean age at primary diagnosis was 12.1 years (range: 7–17.2 years) while mean age at the time of the procedure was 15 years old (range 7–17.9 years), and 10 patients were pre-pubertal at the time of surgery. Patients were affected by: lymphoma (15), osteosarcoma/Ewing sarcoma (7), rhabdomyosarcoma/synovial sarcoma (5), neuroblastic tumor (3), medullary aplasia (2), Chronic granulomatous disease (2), ovarian teratoma (1), medulloblastoma (1).

Main indications for cryopreservation were chemotherapy and radiotherapy (27), chemotherapy for bone marrow transplantation (5), chemotherapy (4), removal of both ovaries for neoplastic risk (2).

The mean operative time was 55 min. Only one procedure was performed in open surgery in a case with bilateral ovarian teratoma. Drainage was not necessary in any of the procedures and patients were discharged on the second post-operative day. In 5 cases the sampling was bilateral. The right ovary was sampled in 28 cases, while the left ovary was selected in the remaining 5 patients.

### 3.4 Center 4

In Center 4, the program of LOTC in girls and adolescents with onco-hematologic diseases started in January 2020. A total of 18 procedures were performed up to December 2022. Patients were diagnosed with leukaemia (4), non-Hodgkin's lymphoma (3), Hodgkin's lymphoma (8), acquired bone marrow aplasia (1), botryoid vaginal rhabdomyosarcoma (1) and Ewing's sarcoma of the pubis (1). Ovarian preservation was performed prior to conditioning for bone marrow transplantation or initiation of gonadotoxic chemotherapy. Age at diagnosis and at intervention varied widely. Patients with leukaemia were diagnosed at birth and preservation surgery was performed on average at 4.86 years (2–10 years). The mean age at diagnosis for the remaining patients was 13.7 years (except for a patient with vaginal rhabdomyosarcoma who was diagnosed at 15 months) and, in this group, surgery was performed at an average of 2.68 months after diagnosis. Only 3 patients were pubertal at the time of surgery.

Depending on the age of each patient and the intraoperative findings (ovarian volume and macroscopic characteristics), it was decided which ovary was sampled and whether to perform an ovarian cortex removal (5) or a total oophorectomy (11), always sampling only one ovary. No bilateral ovarian cortex removal was performed. Seventy-five percent of the procedures were performed on the right ovary. All procedures were approached laparoscopically and none required conversion. Mean operative time was 65 min.

One patient subjected to unilateral ovarian cortex excision required urgent re-laparoscopy due to hemoperitoneum and sudden anemization. Hemoglobin check was not routinely carried out. Patients were discharged from surgery on the same day or the day after the operation. Bone marrow transplantation or chemotherapy was initiated at a mean of 14.7 days after surgery.

No malignant cells were identified at the histopathology evaluation.

## 4 Discussion

This study reports the surgical outcomes of a large multicentric series of young patients including girls and adolescents younger than 17 years of age at diagnosis, who underwent fertility preservation through LOTC at four European high-volume Pediatric Centers.

The population in this study had a high proportion of pediatric-specific diseases such as neuroblastoma, but also common childhood diseases such as leukemia and central nervous system tumors. Apart from leukemia, the most commonly observed pathologies were sarcomas and lymphomas.

Regarding the approach to surgery, there are some differences between Centers.

Bilateral harvesting in Center 1 is opposed to unilateral biopsy in Center 2, 3, and 4, adding the pre-operative ultrasound evaluation.

The drainage was routinely left in place only in Center 2. No minor or major complications were reported in Center 2 and 3, while a postoperative bleeding that needed a re-laparoscopic exploration and a coagulation of the ovarian surface was registered both in Center 1 and 4.

Different techniques for LOTC are reported in literature, such as ovarian cortical biopsies, partial or unilateral total oophorectomy. Some Centers opted for total unilateral ovarian removal, because of the theoretical reduced risk of bleeding and ovarian damage; furthermore, prepuberal ovaries are smaller than their puberal counterpart, so the chances of collecting a sufficient amount of ovarian tissue is greater with a wider removal (2). On the other hand, some patients do not suffer from POF, but rather have a low ovarian reserve and the withdrawal of the entire organ could exacerbate the failure. Moreover, for the patient, it is psychologically easier to accept multiple ovarian sampling than a total oophorectomy (6).

Regardless of the surgical procedure preferred, the laparoscopic approach is the most common in children and adolescents. However, the minimal invasive technique can increase the risk of post operative bleeding and anemia. There is no universal consensus on which is the optimal and safest hemostatic procedure. Electrocauterization is arguably the fastest and most secure way; at the same time, it has been reported that repeated cauterization could cause an important reduction in the ovarian reserve, due to irreversible vessel damage and the subsequent tissue reaction. The intracorporeal suture of the ovarian bed is undoubtedly the best option in terms of preservation of the remaining ovarian tissue; nevertheless, this is difficult to accomplish in a short time and it requires a long hemostatic time, so is unsuitable for emergency procedure (7).

The three most common techniques for ovarian tissue cryopreservation are the following: vitrification, slow-freezing, and ultra-rapid freezing (8). The slow-freezing technique, used for the ovarian tissue cryopreservation of this series of patients, was firstly described by Behrman and Savada in 1966 (9–11). This technique consists of a gradual cooling of the sample using liquid nitrogen and aims to reduce ovarian toxicity related to a lower concentration of the cryoprotectants used. The cryoprotectant is added by sequential dilution in two steps, with minor modifications. The ovarian tissue fragments are incubated at low temperatures between 2 and 10 degrees centigrade.

To date, transplantation of cryopreserved ovarian tissue has resulted in births of at least 130 children but data on transplantation of ovarian tissue removed before puberty are scarce. Currently in the literature, 4 patients under 15 years of age at the time of OTC have used their cryopreserved ovarian tissue (12).

LOTC was firstly described by Hovatta et al. in 1996 (13). However, the first publication showing the functionality of ovarian tissue cryopreserved before puberty was published in 2012. It showed a patient with sickle cell disease in whom ovarian freezing was carried out at age 10 before a myeloablative conditioning regimen followed by allogeneic HSCT. Twenty-seven months after HSCT, the patient and her mother returned to request an ovarian tissue autotransplantation to induce spontaneous puberty because she had POF. A heterotopic autotransplantation of three fragments of ovarian cortex succeeded in inducing puberty with the onset of the first menstrual period 8 months after the ovarian transplantation (14).

The following year, Ernst et al, confirmed that ovarian cortex transplantation could induce puberty in a patient who was 9 years old at the time of OTC prior to treatment for Ewing's sarcoma (15).

The first birth obtained after transplantation of ovarian tissue cryopreserved before menarche was reported in 2015 (16). This patient underwent OTC before HSCT for the treatment of sickle cell disease. At the time of the ovarian cryopreservation, she was 14 years old and she had still not had a menstrual period. Ten years later, the patient had a POF and requested an ovarian tissue transplantation to restore her fertility. She had her first menstrual period 5 months after the procedure and became spontaneously pregnant more than 2 years after the transplantation, giving birth to a healthy boy.

The youngest patient at the time of ovarian freezing, who gave birth after ovarian cortex transplantation, was 9 years old at the time of tissue retrieval. She had beta-thalassemia treated by HSCT preceded by a gonadotoxic conditioning regimen before HSCT (17).

These rare publications confirm that beyond the age of 9, ovarian tissue can be functional after transplantation (4). It remains to be determined whether this can be applied for girls younger than 9 years old.

The main limitation of the present study is its focus only on the short-term surgical outcomes, examining the incidence of short-term postoperative complications in the first thirty days after surgery and in the following six months, without examining the outcomes in terms of endocrine function and fertility of our patients. Future studies related to these variables will be necessary to understand the global impact of LOTC on the endocrine and fertility outcomes of oncological patients.

Another limitation of the present study is represented by the heterogeneity of the laparoscopic technical aspects of each Centers, such as the diameter of the trocar used for the first access and the time frame in which the data were collected (2 years for Center4, 11 years for Center3, 20 years for Center1 and 2). Moreover, the global data collection of patients submitted to surgery over a period of approximately 20 years could be burdened by biases linked to technical and technological improvements in minimally invasive surgery, although both the dimensions of the trocars and the type of surgical instruments used for LOTC have been standardized in each Center for the present series of patients. Due to the young age of the patients involved, the routine use of a 5 mm optic trocar for the first access should be considered in further studies.

Regarding ethical and medico-legal issues, a fertility preservation technique should ideally be evidence-based, should not create false hopes, unrealistic expectations or harm the patient.

The problem with cryopreservation is the experimental label which has not yet been removed in many countries, especially because the evaluation of effectiveness takes many years and because of the risk of reimplantation of cancer cells. It is well known, for patients with acute lymphoblastic leukemia or lymphoma, that the ovarian stroma may contain malignant cells. Rosendahl et al. reported 26 cases of ovarian tissue taken from woman with diagnosis of leukemia and, even if immunohistochemistry was unable to detect malignant cells in the cortex, in most of the sampling, it was possible to discover potential tumoral cells using PCR (18).

Although, up to date, there is no evidence that these cells are viable or that the disease could relapse once they are reimplanted, in these cases the procedure is not recommended. There are now numerous studies about alternative methods for fertility restoration in patients with residual malignant cells in the stroma, such as follicle isolation, *in vitro* follicle maturation or *in vitro* fertilization.

If these factors can create some doubts, it is necessary to consider that many studies attest distress, anxiety and depression related to future infertility. Cryopreservation is the only way for pediatric surgeons to preserve young patients' fertility in prepubertal age. We must also consider the positive effect that thinking about the future provides for patients dealing with an oncologic pathology.

As for intra- or post-operative complications the rate remains low in most of the studies.

Beckmann et al., demonstrated that the rate of complications for ovarian tissue removal and transplantation is so low that it can be compared to those from standard laparoscopy (19).

Furthermore, laparoscopy offers a great advantage in young cancer patients as it allows a direct oncological staging thanks to the exploration of the entire abdomen. Finally, the advantage of the pediatric age is precisely the question of time, that is the fact that we do not know how much progress science will have made when this tissue will be requested by patients in 10 or 20 years, when new therapeutic possibilities might be available.

Within a few years, long-term data regarding this cohort of patients will be available, which will add to the short-term surgical data, already presented in the present study, information about the impact of the LOTC on long-term fertility and endocrine outcomes.

## 5 Conclusions

LOTC is a promising modality to preserve fertility in pediatric population submitted for gonadotoxic therapies. Even if there are different techniques and approaches, the procedure is safe, easy to perform and reliable, and offers a minimal invasive approach in high-risk patients, without delaying the onset of life saving chemo or radiotherapy. This series of 311 patients from different European Centers was comparable in terms of technical approaches and outcomes. A team of specialized surgeons is needed to grant and develop a standardized procedure. Future studies including endocrine and fertility outcomes will clarify the global and long-term clinical significance of LOTC.

## Data Availability

The raw data supporting the conclusions of this article will be made available by the authors, without undue reservation.
